# Supplementary material for: Development of a Tet-On Inducible Expression System for the Anhydrobiotic Cell Line, Pv11
Source: Insects. 2020 Nov 11;11(11):781. doi: 10.3390/insects11110781 (PMC7696976; doi:10.3390/insects11110781)
Supplement: Supplementary file 1 [file insects-11-00781-s001.zip › insects-975884-supplementary/insects-975884-supple-conversion/insects-975884-supple-Figures and Tables proof.docx]

**Figure S1.** The map of pPv121-MCS vector. The multiple cloning site of the vector includes BamHⅠ, HindⅢ, XhoⅠ and SacⅡ sites.

**Figure S2.** Validation of Western blotting analysis. (**A**) Protein blotted membrane shown in Fig. 2 was stained with ponceau S (left panel) and immunoreacted with anti-GFP antibody (right panel). The molecular weight of AcGFP1 is 27 kDa (red arrow). (**B**) Protein blotted membrane shown in Fig. 3 was stained with ponceau S (left panel) and immunoreacted with anti-His-tag antibody (right panel). The molecular weight of AMV RTα is 63 kDa (red arrow). The relative intensity of bands was measured with the image lab software (Bio-Rad, version 6.1). Relative intensities of the band for AcGFP1 or AMV RTα compared with the Dox-treated cells transfected with pTetO-202bp-AcGFP1 or pTetO-202bp-AMV RTα in IPL, respectively, are indicated beneath each lane. The three types of marker were used (Marker 1, Cat#1610374 by Bio-Rad; Marker 2, Cat#LC5602 by Thermo Fisher Scientific; Marker 3, Cat#02525 by Nacarai Tesque). ND, not detected.


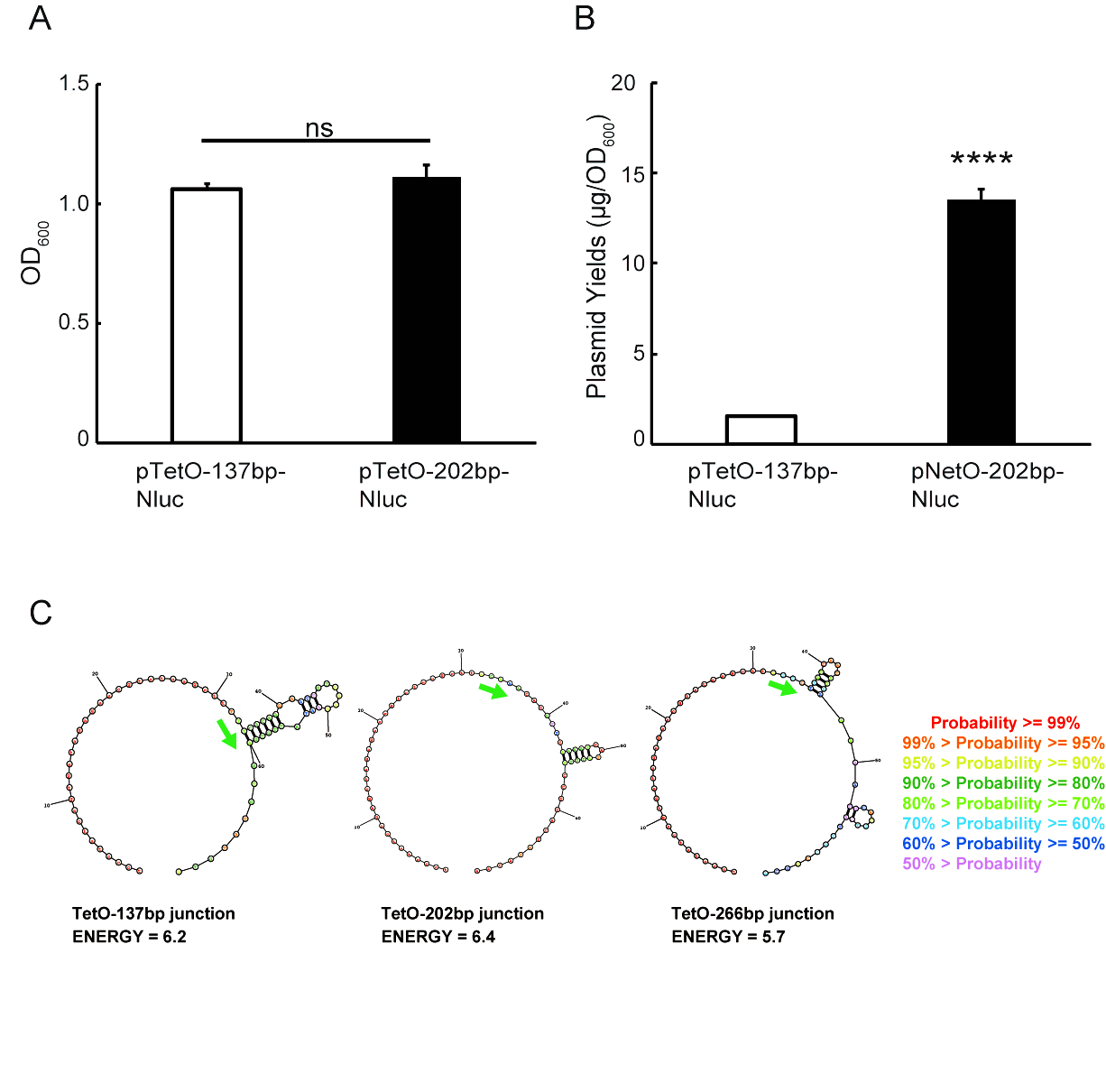


**Figure S3.** The 137bp fragment from the 121-promoter and its secondary structure. (**A**) Optical density (OD) of *E. coli* transformed with pTetO-137bp-Nluc and pTetO-202bp-Nluc. The transformants were cultured in LB medium containing ampicillin at 37℃ for 16 hours, then OD_600_ was measured. (**B**) Yields of the pTetO-137bp-Nluc and pTetO-202bp-Nluc plasmids. The plasmids were purified, and the concentration was measured. Normalized values are expressed as mean ± standard deviation (SD). *****p<0.0001*; ns, not significant; n=3 in each group. (**C**) Secondary structure of 69bp junction regions of TetO area (31bp) and 137bp-, 202bp- and 266bp- fragments. The secondary structures of TetO and each fragment region was predicted by MaxExpect algorithm [1] for DNA sequences with remaining parameters set to default. Green arrows define the starting point for each fragment region. The TetO area has no possible secondary structures, while the sequences of the various fragments tested may fold into different stem-loop structures, while might affect plasmid function melting to single strands, as in replication. According to free energy values, The TetO-137bp stem-loop is most stable.

**Table S1.** Primers for the construction of AcGFP1, Nluc and AMV RTα expression vectors.

| **Target Fragment-of-Interest** | **Forward Primer** | **Reverse Primer** |
| --- | --- | --- |
| AcGFP1 | CACTTCCTACCCTCGTAAAGTCGACCAACATGG  TGAGCAAGGGCGCC | TGATATCCGGCCGATCGATAGATCTTTAC  TTGTACAGCTCATCCATGCCG |
| 202bp-fragment | TCAGTGATAGAGAACGTATAAGCTTACAAACAC  GTTCAAAATCATATTTTC | GCCCTTGCTCACCATGTTGGTCGACTTTT  TTCAGAAAATATTTTCTTTTGTC |
| 266bp-fragment | TCAGTGATAGAGAACGTATAAGCTTATTCTTATC  AATATAAAAAATGCATGTC | GCCCTTGCTCACCATGTTGGTCGACTTTT  TTCAGAAAATATTTTCTTTTGTC |
| Nluc for pTRE3G vector | AAGAAAATATTTTCTGAAAAAAGTCGACCAAC  ATGGTCTTCACACTCGAAG | TGATATCCGGCCGATCGATAGATCTTTAC  GCCAGAATGCGTTC |
| Tet-On 3G | AAGAAAATATTTTCTGAAAAAAGGATCCCAAC  ATGTCTAGACTGGACAAGAGC | TAGGCTTACCTTCGAACCGCGGTTACCC  GGGGAGCATGTC |
| Nluc for 121-promoter vector | CTTTCGTCTTCAAGAATTCCTCGAGCTTCAATTA  TGATACATGAATAAACAAAATATTAAAG | GAGTGTGAAGACCATGTTGGTCGACTTT  TTTCAGAAAATATTTTCTTTTGTC |
| 632bp-fragment | GCTAGCCTCGAGGATATCAAGATCTGGAAAATCACATGGACATC | CCAACAGTACCGGATTGCCAAGCTTTTTT  TTCAGAAAATATTTTCTTTTGTC |
| AMV RTα for the 121 vector | AAGAAAATATTTTCTGAAAAAAGGATCCCAAC  ATGACTGTTGCGCTACATCTG | AAGAAAATATTTTCTGAAAAAAGGATCC  CAACATGACTGTTGCGCTACATCTG |
| AMV RTα for　pTRE3G vector | AAGAAAATATTTTCTGAAAAAAGTCGACCAAC  ATGACTGTTGCGCTAC | TGATATCCGGCCGATCGATAGATCTTTAG  TGGTGATGGTGGTG |

The primers were designed using the NEBuilder Assembly Tool Verdion1 (https://nebuilderv1.neb.com/).

**Table S2.** The combination of transfected vectors in each experiment.

| **Experiment** | **Sample Name in Figures** | **Transfected Vectors** | | |
| --- | --- | --- | --- | --- |
|  |  | **1** | **2** | **3** |
| Luciferase assay in  Figure 1A | Luc | pNL1.1 | pPv121-TetOn 3G | pPv632bp-luc2 |
|  | TetO-Luc | pTetO-Nluc | pPv121-TetOn 3G | pPv632bp-luc2 |
|  | TetO-CMV-Luc | pTetO-CMV-Nluc | pPv121-TetOn 3G | pPv632bp-luc2 |
|  | TetO-202bp-Luc | pTetO-202bp-Nluc | pPv121-TetOn 3G | pPv632bp-luc2 |
|  | TetO-266bp-Luc | pTetO-266bp-Nluc | pPv121-TetOn 3G | pPv632bp-luc2 |
|  | 121-promoter-Luc | pPv121-Nluc | pPv121-TetOn 3G | pPv632bp-luc2 |
| Expression of AcGFP1 in  Figure 2A and 2B | Empty vector | pPv121-MCS | - | - |
|  | pTetO-202bp-AcGFP1 | pTetO-202bp-AcGFP1 | pPv121-TetOn 3G | - |
|  | pPv121-AcGFP1 | pPv121-AcGFP1 | pPv121-MCS | - |
| Expression of AMV RTα in  Figure 3A and 3B | Empty vector | pPv121-MCS | - | - |
|  | pTetO-202bp-AMV RTα | pTetO-202bp-AMV RTα | pPv121-TetOn 3G | - |
|  | pPv121-AMV RTα | pPv121-AMV RTα | pPv121-MCS | - |

**Table S3.** Statistical analysis of luciferase activities of the cells transfected with the corresponding vectors in Figure 1A.

|  |  |  | **IPL-41** | | | | | | | | | | **Trehalose** | | | | | | | | | |
| --- | --- | --- | --- | --- | --- | --- | --- | --- | --- | --- | --- | --- | --- | --- | --- | --- | --- | --- | --- | --- | --- | --- |
|  |  |  | pNluc | | Nonsense | | CMV | | 202bp | | 266bp | | pNluc | | Nonsense | | CMV | | 202bp | | 266bp | |
|  |  | Dox | - | + | - | + | - | + | - | + | - | + | - | + | - | + | - | + | - | + | - | + |
| IPL-41 | pNluc | - |  | ns | ns | ns | ns | ns | ns | **** | **** | **** | ns | ns | ns | ns | ns | ns | ns | **** | ** | **** |
|  |  | + |  |  | ns | ns | ns | ns | ns | **** | **** | **** | ns | ns | ns | ns | ns | ns | ns | **** | ** | **** |
|  | Nonsense | - |  |  |  | ns | ns | ns | ns | **** | **** | **** | ns | ns | ns | ns | ns | ns | ns | **** | ** | **** |
|  |  | + |  |  |  |  | ns | ns | ns | **** | **** | **** | ns | ns | ns | ns | ns | ns | ns | **** | ** | **** |
|  | CMV | - |  |  |  |  |  | ns | ns | **** | **** | **** | ns | ns | ns | ns | ns | ns | ns | **** | ** | **** |
|  |  | + |  |  |  |  |  |  | ns | **** | *** | **** | ns | ns | ns | ns | ns | ns | ns | **** | * | **** |
|  | 202bp | - |  |  |  |  |  |  |  | **** | *** | **** | ns | ns | ns | ns | ns | ns | ns | **** | * | **** |
|  |  | + |  |  |  |  |  |  |  |  | **** | **** | **** | **** | **** | **** | **** | **** | **** | ns | **** | ns |
|  | 266bp | - |  |  |  |  |  |  |  |  |  | **** | **** | **** | **** | **** | **** | *** | ** | **** | ns | **** |
|  |  | + |  |  |  |  |  |  |  |  |  |  | **** | **** | **** | **** | **** | **** | **** | **** | **** | ns |
| Trehalose | pNluc | - |  |  |  |  |  |  |  |  |  |  |  | ns | ns | ns | ns | ns | ns | **** | ** | **** |
|  |  | + |  |  |  |  |  |  |  |  |  |  |  |  | ns | ns | ns | ns | ns | **** | ** | **** |
|  | Nonsense | - |  |  |  |  |  |  |  |  |  |  |  |  |  | ns | ns | ns | ns | **** | ** | **** |
|  |  | + |  |  |  |  |  |  |  |  |  |  |  |  |  |  | ns | ns | ns | **** | ** | **** |
|  | CMV | - |  |  |  |  |  |  |  |  |  |  |  |  |  |  |  | ns | ns | **** | ** | **** |
|  |  | + |  |  |  |  |  |  |  |  |  |  |  |  |  |  |  |  | ns | **** | * | **** |
|  | 202bp | - |  |  |  |  |  |  |  |  |  |  |  |  |  |  |  |  |  | **** | ns | **** |
|  |  | + |  |  |  |  |  |  |  |  |  |  |  |  |  |  |  |  |  |  | **** | ns |
|  | 266bp | - |  |  |  |  |  |  |  |  |  |  |  |  |  |  |  |  |  |  |  | **** |
|  |  | + |  |  |  |  |  |  |  |  |  |  |  |  |  |  |  |  |  |  |  |  |

The red squares indicate leaky expression of 266bp fragment. Statistical analysis was performed by Tukey test as a post hoc test for three-way ANOVA. **p* < 0.05; ***p* < 0.01; ****p* < 0.001; *****p* < 0.0001; ns, not significant; n = 5 in each group.

**Table S4.** Statistical analysis of reverse transcript activities of the cells transfected with the corresponding vectors in Figure 3B.

|  |  |  | IPL-41 | | | | Trehalose | | | |
| --- | --- | --- | --- | --- | --- | --- | --- | --- | --- | --- |
|  |  |  | Empty | | 202bp | | Empty | | 202bp | |
|  |  | Dox | - | + | - | + | - | + | - | + |
| IPL-41 | Empty | - |  | ns | ns | * | ns | ns | ns | ** |
|  |  | + |  |  | ns | ns | ns | ns | ns | * |
|  | 202bp | - |  |  |  | * | ns | ns | ns | ** |
|  |  | + |  |  |  |  | * | * | ns | ns |
| Trehalose | Empty | - |  |  |  |  |  | ns | ns | ** |
|  |  | + |  |  |  |  |  |  | ns | ** |
|  | 202bp | - |  |  |  |  |  |  |  | * |
|  |  | + |  |  |  |  |  |  |  |  |

Statistical analysis was performed by Tukey test as a post hoc test for three-way ANOVA. **p* < 0.05; ***p* < 0.01; ns, not significant; n = 3 in each group.

Reference

[1] Reuter, J.S. and Mathews, D.H. "RNAstructure: software for RNA secondary structure prediction and analysis." *BMC Bioinformatics*, **2010**, *11*, 129.
